# The Protective Effect of *Agaricus blazei* Murrill, Submerged Culture Using the Optimized Medium Composition, on Alcohol-Induced Liver Injury

**DOI:** 10.1155/2014/573978

**Published:** 2014-07-10

**Authors:** Hang Wang, Gang Li, Wenyu Zhang, Chunchao Han, Xin Xu, Yong-Ping Li

**Affiliations:** ^1^School of Pharmacy, Shandong University of Traditional Chinese Medicine, Jinan 250355, China; ^2^Department of Neurosurgery, Tangdu Hospital, Fourth Military Medical University, Xi'an 710032, China; ^3^Department of Vascular and Endocrine Surgery, Xijing Hospital, Fourth Military Medical University, Xi'an 710032, China

## Abstract

*Agaricus blazei* Murrill (ABM), an edible mushroom native to Brazil, is widely used for nonprescript and medicinal purposes. Alcohol liver disease (ALD) is considered as a leading cause for a liver injury in modern dietary life, which can be developed by a prolonged or large intake of alcohol. In this study, the medium composition of ABM was optimized using response surface methodology for maximum mycelial biomass and extracellular polysaccharide (EPS) production. The model predicts to gain a maximal mycelial biomass and extracellular polysaccharide at 1.047 g/100 mL, and 0.367 g/100 mL, respectively, when the potato is 29.88 g/100 mL, the glucose is 1.01 g/100 mL, and the bran is 1.02 g/100 mL. The verified experiments showed that the model was significantly consistent with the model prediction and that the trends of mycelial biomass and extracellular polysaccharide were predicted by artificial neural network. After that, the optimized medium was used for the submerged culture of ABM. Then, alcohol-induced liver injury in mice model was used to examine the protective effect of ABM cultured using the optimized medium on the liver. And the hepatic histopathological observations showed that ABM had a relatively significant role in mice model, which had alcoholic liver damage.

## 1. Introduction

Alcoholic liver disease (ALD) has become a common global healthcare problem, which is due to excessive alcohol intake for a long duration. Liver is the leading organ for synthesizing vitals, metabolizing ingesta, and detoxifying noxious substances. People who drink a lot are more likely to suffer from ALD including steatohepatitis, hepatic fibrosis, and cirrhosis [1, 2]. Hepatic tissue can be damaged by dangerous byproducts of alcohol breakdown like acetaldehydes, which can react with cellular proteins to generate adducts. Therefore, it is particularly important to find a kind of natural and low-toxic medicine in treatment of alcoholic liver disease.

Mushrooms are defined as macrofungi with distinctive and visible fruiting bodies that may grow above or below ground [3]. Nutritional value of edible mushrooms is due to high protein, fiber, vitamin and mineral contents, and a low-fat level [4–7]. Owing to their attractive taste, aroma, and nutritional values, edible mushrooms were used as food and medicine for centuries [8, 9], including biologically active polysaccharides in the fruiting bodies and bioactive compounds in submerged broth. And some mushrooms popular in the Far East have been reported to have medicinal value, including antitumor, antiviral, and hypolipidemic effects [3, 10, 11].

*Agaricus blazei* Murrill (ABM), a mushroom native to Brazil, is a basidiomycete brown fungus, which is widely used for nonprescript, medicinal purposes, both as an edible mushroom and in the form of extracts. Due to alleged health effects, ABM was brought to Japan and is widely used today in oriental countries both as an edible mushroom, considered a functional food, and as a natural therapy in the form of a medicinal extract mostly for prevention and treatment of cancer [12]. And many bioactive components of ABM have been studied showing that it has relatively notable pharmacological effects, especially the polysaccharides. To reduce the cost and improve the productivity, numerous researchers have studied the production of the mycelium and polysaccharide by submerged fermentation of ABM [13–17]. There are a good number of advantages of submerged culture including higher polysaccharide and mycelium production in a more compact space and shorter time [18] and as an alternative for efficient production of polysaccharide with similar biological activity [19].

Consequently, in the present paper, the most optimum conditions for maximum mycelia biomass and extracellular polysaccharide production were investigated in ABM using response surface methodology and the artificial neural network (ANN). What is more, this study investigated protective effects of oral administration of ABM on alcoholic liver damage.

## 2. Materials and Methods

### 2.1. Microorganism

ABM was obtained from the Center for Culture Collection of Pharmaceutical Microorganisms, China. The culture was maintained in potato dextrose agar (PDA) slant containing (per liter) 200.0 g potato juice, 20.0 g glucose, and 20.0 g agar and incubated at 25°C for 7 days. The slants were subcultured every three months and then stored at 4°C.

### 2.2. Flask Culture

Potato dextrose broth (PDB) was prepared as follows: 200.0 g potatoes were cut into 1 cm^3^ pieces and boiled in 500 mL of water for 30 min. Simultaneously, 20.0 g wheat bran was also boiled in 500 mL of water for 30 min and then the extracts of potatoes and bran were collected by filtration through gauze. Next, 20.0 g glucose, 1.0 g KH_2_PO_4_, 1.5 g MgSO_4_ *·*7H_2_O, and water were added to the extracts to 1 L total volume. Then the medium was autoclaved at 121°C for 30 min. The flask culture experiments were performed in 250 mL flasks containing 150 mL of fermentation medium, which was inoculated with 10% (v/v) of the seed culture. Finally, the flasks were inoculated and incubated at 25°C for 6 days in a static condition.

### 2.3. Response Surface Methodology for Optimizing Medium Components

Response surface methodology (RSM) was used to investigate the optimum concentration of the variables (concentration of glucose, potato, and bran extract) on the maximum yield of mycelial biomass growth and EPS production from ABM. The software Design-Expert 7.1 Trial was applied in the experimental design, data analysis, and quadratic equation construction. The experimental design was a Box-Behnken design with three key factors and three levels (Table 1). Triplicates at the center (−1, 0, and 1) of the design were intended to allow the estimation of the pure error sum of squares. For statistical calculation, the independent variables were coded according to the following equation:

(1) $$\begin{array}{c} \;x_{i}=\frac{X_{i}-X_{0}}{\Delta X_{i}},\;i=1,2,3, \end{array}$$

where *X*_*i*_ is the real value of an independent variable, *X*_0_ is the real value of the independent variable on the center point and Δ*X*_*i*_ is the step change value, and *x*_*i*_ represents the coded values for *X*_*i*_. As shown in Table 1, these three independent variables were coded as *X*_1_, *X*_2_, and *X*_3_, respectively.

The behavior of the system is explained by the following second-degree polynomial equation:

(2) $$\begin{array}{c} Y=b_{0}+\sum b_{i}X_{i}+\sum b_{ii}X_{i}^{2}+\sum b_{ij}X_{i}X_{j}, \end{array}$$

where *Y* is the predicted response value, *b*_0_ is the intercept term, *b*_*i*_ is the linear term, *b*_*ii*_ is the squared term, *b*_*ij*_ is the interaction term, and *X*_*i*_ and *X*_*j*_ are the coded level of independent variables.

Statistical analysis of the model was performed to evaluate ANOVA. The goodness of fit of the polynomial model equation was expressed by Fisher's *F*-test, the coefficient of determination *R* ^2^. And the surface and contour plots express the fitted polynomial equation, which can visualize the relationship between experimental levels of each factor and the response to deduce the optimum conditions [20].

### 2.4. Estimation of the Mycelium Dry Weight and Extraction of EPS

The mycelial biomass of ABM was measured by filtering the fungal culture through the gauze until a clear filtrate was obtained. The obtained mycelium was washed three times with distilled water, dried to constant weight at 60°C, and weighed. Then EPS was precipitated from the culture filtrate, mixed with five volumes of 95% (v/v) ethanol, and left to stand overnight at 4°C to precipitate crude EPS. The precipitated EPS was centrifuged by centrifugation at 4500 rpm for 5 min and the supernatant was concentrated so that ethanol could be recovered. The centrifugated precipitate was resuspended in an equal volume of 75% ethanol [21] to remove oligosaccharides and centrifuged again. The precipitated EPS was dried to constant weight at 60°C to remove residual ethanol and weighed.

### 2.5. Assay of Extracellular Polysaccharide from ABM

The dry EPS was ground into fine power and estimated by the phenol-sulfuric acid colorimetric assay [22]. In brief, 20 mg EPS was fully dissolved in 100 mL of double distilled water and mixed evenly. 0.4 mL of the above solution was added in 1.6 mL double distilled water to 2 mL total volume, followed by addition of 1 mL of phenol and 5 mL of sulfuric acid, and then the polysaccharide content of the mixture was determined spectrophotometrically at 490 nm by using glucose as standard.

### 2.6. Optimization of Media Components by Artificial Neural Network

The artificial neural network (ANN) is a collection of mathematical and statistical techniques useful for analyzing the effects of several independent variables. Application of ANN has been considered as a promising tool because of their simplicity towards simulation, prediction, and modeling. A back-propagation algorithm is a multilayer feed-forward ANN, which can train and then evaluate the system performance using an adaptive gradient learning rule [23–25]. In this paper, experimental values of three variables, mycelial dry weight, and extracellular polysaccharide in Table 2 were normalized in the range of 0 to 1. Then three neurons in the input layer, three in a hidden layer, and one in the output layer using the tanh transfer function were used to predict the yield of mycelial biomass and EPS, respectively (Matlab 7.1 software).

### 2.7. Evaluation of ABM against Alcohol-Induced Liver Injury in Mice

#### 2.7.1. Preparation of ABM

The optimized medium was used for the submerged culture of ABM. After the completion of the fermentation culture, ABM was filtered with gauze after submerged culture. Then the filtrate was collected as ABM fermentation broth (ABM-fb). Fermentation mycelia were watered twice with distilled water and homogenized with organization pounding machine (ABM-fm). ABM fermentation product (ABM-fp), including fermentation broth and mycelia, was homogenized with organization pounding machine. The above samples were stored at 4°C in bottles and kept sterile until instilled intragastrically in mice.

#### 2.7.2. Animals Model and Liver Histology

Male Kunming mice were obtained from Lukang Animal Pharmaceutical Co., Ltd. (Shandong, China) and acclimated for 1 week in the animal experimental research laboratory. Animals were randomly divided into 5 groups with 10 mice per each. The normal control group was fed with normal diet and water. The ethanol control group was fed with Chinese white liquor except the normal diet. The other three experimental groups were also fed with Chinese white liquor and were simultaneously fed with ABM fermentation broth (ABM-fb), ABM fermentation mycelia (ABM-fm), and ABM fermentation products (ABM-fp), respectively. All animals were maintained on the treatments for a total of 4 weeks. Liver samples were collected and fixed in formalin for histology study. And formalin-fixed paraffin tissue sections were processed for staining with hematoxylin and eosin and then studied by light microscopy.

### 2.8. Statistical Analysis

The data were analyzed using SPSS and expressed as means ± standard deviation (SD). Differences were considered statistically significant when *P* < 0.05 by one-way analysis of variance (ANOVA).

## 3. Results and Discussions 

The Box-Behnken design matrix was used to determine the effects of the three independent variables including concentration of potato extract (*X*_1_), concentration of glucose (*X*_2_), and concentration of wheat bran extract (*X*_3_) on the yield of mycelial biomass and extracellular polysaccharide. The experimental and predictive values of responses (yield of the extracellular polysaccharides and mycelial dry weight) under different treatment conditions are presented in Table 2.

### 3.1. Optimization of the Yield of Mycelial Biomass by RSM

The predicted response *Y*_biomass_ for the mycelial biomass in terms of coded unit was obtained as follows:

(3) Ybiomass=0.84+0.091X1+0.081X2−0.010X3 +0.010X2X3+0.027X12 −0.0014X22−0.018X32,

where *Y*_biomass_ is the response in terms of g/100 mL of mycelial biomass, where *X*_1_, *X*_2_, and *X*_3_ are independent variables in coded units containing concentration of potato extract, concentration of glucose, and concentration of wheat bran extract, respectively. Statistical testing of the regression model was done in the form of an *F*-test and the analysis of variance (ANOVA), which is required to test the significance and adequacy of the model (Table 3).

In this study, ANOVA of the regression model demonstrated that the model was highly significant for the mycelial biomass yield, as was evident from the calculated *F*-value (model = 216.98) with a very low probability value (*P* > *F*) < 0.0001 [26]. Values of “*P* > *F*” less than 0.05 indicate that model terms are significant. In this case, the *P* values were much less than 0.05, indicating that all these variables were more significant. The *P* value was used as a tool to check the significance of each of the coefficients, which are necessary to understand the pattern of mutual interactions between the test variables. The correlation measure for testing the goodness of the model was the coefficient of determination (*R* ^2^), which should be closer to 1. In the present study, the *R* ^2^ was 0.9964, much closer to 1. The predictive *R* ^2^ (Pred-*R* ^2^) of 0.9429 was in reasonable agreement with the adjustable *R* ^2^ (adj-*R* ^2^) of 0.9918. The adequate precision was also used to measure the ratio of signal to noise and it was generally desired to be greater than 4 [27]. The value of adequate precision was 56.205 suggesting that the model was of an adequate signal and could be used to navigate the design space. Hence, the regression model given in (3) is a good prediction of the experimental results and the factor effects are real.

**Figure 1:**
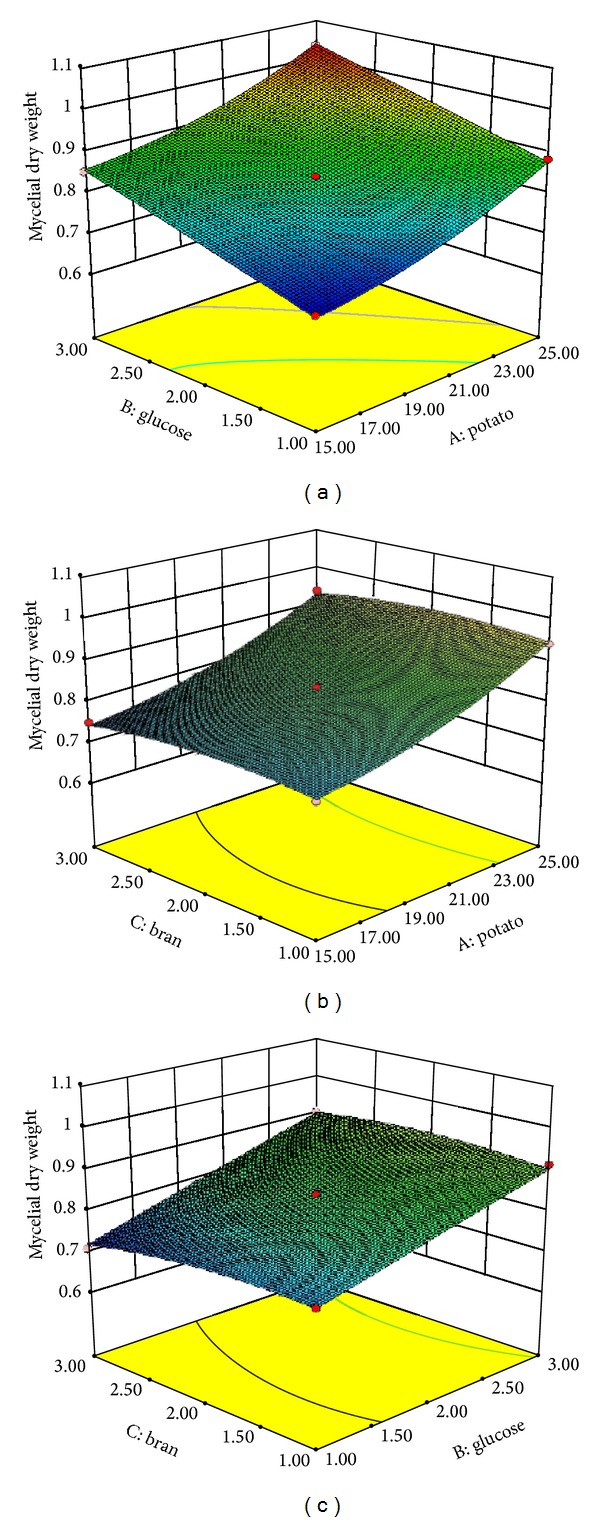
The 3D-plot and 2D-projection of response surface represent the interaction between two factors in the yield of mycelial biomass (g/100 mL) by keeping the other two factors constant: (a) potato and glucose (g/100 mL), (b) potato and bran (g/100 mL), and (c) glucose and bran (g/100 mL).

The graphical representations of the regression equation (3), called the response surfaces and the contour plots, are presented in Figures 1(a), 1(b), and 1(c). The three-dimensional response surface (3D-surface) plot and two-dimensional response projection (2D-projection) were able to visually show the response over a region of interesting factor levels, the relationship between experimental levels of each variable, and the response and the type of interactions between test variables so that the optimum conditions for mycelium production could be deduced [27]. Each plot shows the effect of two independent variables varying within the experimental range of mycelial biomass.  Figure 1(a) shows the effect of potato and glucose concentration on mycelial dry weight. A quadratic effect of potato and glucose concentration on the response was observed. The 3D-plot showed evidence that the yield of mycelial biomass increased upon increasing potato from 15 g/100 mL to 25 g/100 mL and glucose from 1 g/100 mL to 3 g/100 mL, respectively.  Figure 1(b) shows the effect of potato and bran concentration on mycelial dry weight. Unlike Figure 1(a), the yield of biomass decreased with the increase of bran.  Figure 1(c) shows the effect of glucose and bran concentration on mycelial dry weight. A quadratic effect of glucose and bran in the response was observed. And a similar trend of Figure 1(b) was also observed.

### 3.2. Optimization of the Yield of EPS by RSM

Using the designed experimental data (Table 2), the polynomial model for EPS yield *Y*_EPS_ in terms of coded unit was expressed as follows:

(4) YEPS=0.26+0.019X1−0.012X2−0.054X3 −0.00025X1X2+0.008X2X3−0.0091X12 +0.0024X22+0.027X32,

where *Y*_EPS_ is the response in terms of g/100 mL of the yield of extracellular polysaccharide, where *X*_1_, *X*_2_, and *X*_3_ are independent variables in coded units containing concentration of potato extract (g/100 mL), concentration of glucose (g/100 mL), and concentration of wheat bran extract (g/100 mL), respectively.

Table 3 showed the analysis of variance (ANOVA) for response surface quadratic model and the statistical significance of (4) that was checked by *F*-test. The ANOVA of the regression model demonstrated that the model was highly significant for the extracellular polysaccharide, as was evident from the calculated *F*-value (model = 90.04) with a very low probability value (*P* > *F*) < 0.0001 [26]. And a high degree of precision, reliability, and high degree of correlation between the observed and predicted values were demonstrated by the values of correlation coefficient *R* ^2^ (0.9914), adj-*R* ^2^ (0.9804), and Pred-*R* ^2^ (0.8630) [28]. In addition, the value of adequate precision was 33.529 suggesting that the model was of an adequate signal and could be used to navigate the design space. Hence, the regression model given in (4) is a good prediction of the experimental results, and the factor effects are real.

**Figure 2:**
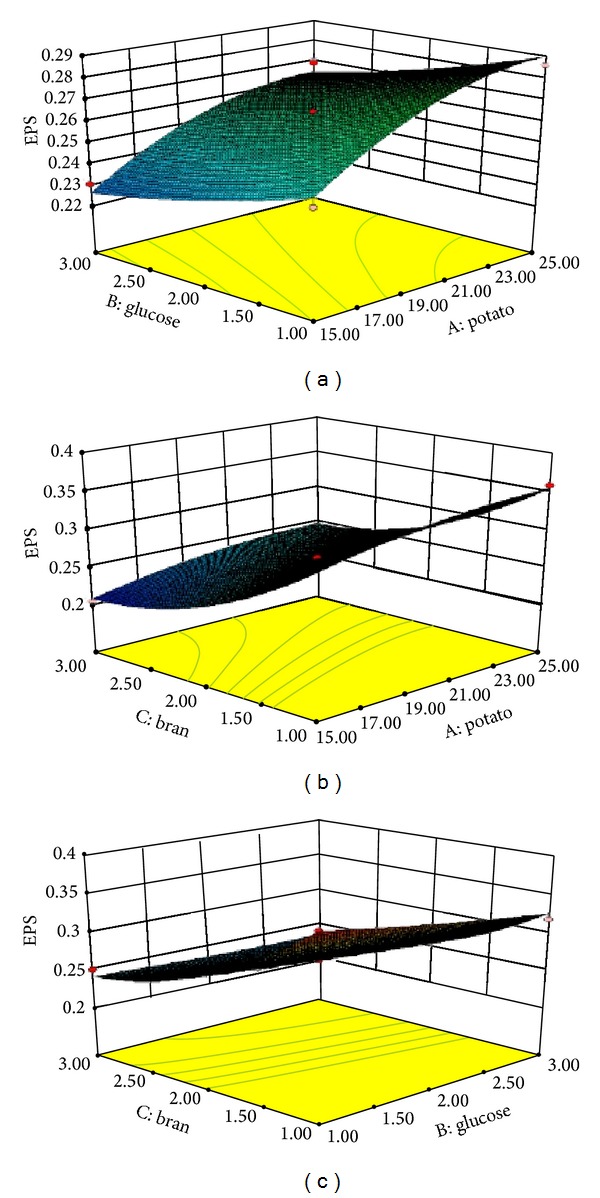
The 3D-plot and 2D-projection of response surface represent the interaction between two factors in the yield of extracellular polysaccharide (EPS) (g/100 mL) by keeping the other two factors constant: (a) potato and glucose (g/100 mL), (b) potato and bran (g/100 mL), and (c) glucose and bran (g/100 mL).

The graphical representations of the regression equation (4), called the response surfaces and the contour plots, are presented in Figures 2(a), 2(b), and 2(c). The 3D-surface plot and 2D-projection could visually show the interaction between the potato (*X*_1_), glucose (*X*_2_), and wheat bran (*X*_3_). The yield of EPS increased sharply with the increase of potato and glucose in Figure 2(a). However, Figure 2(b) showed that the yield of extracellular polysaccharide increased with the increase of potato and the decrease of bran. The maximum yield of EPS was presented when the potato was 25 g/100 mL and the bran was 1 g/100 mL in Figure 2(b).  Figure 2(c) shows the effect of glucose and bran concentration on the extracellular polysaccharide. And the 3D-surface plot and 2D-projection showed the same trend as that in Figure 2(a).

### 3.3. Optimization of the Mycelial Dry Weight and Polysaccharide Production by ANN

**Figure 3:**
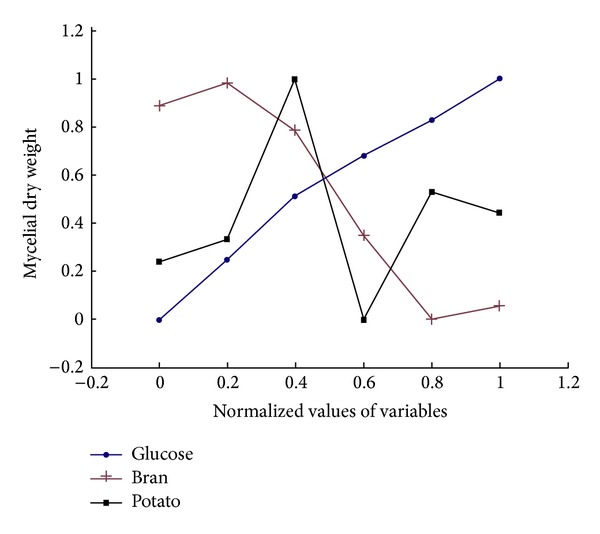
Predicted trend of mycelial dry weight by ANN.

**Figure 4:**
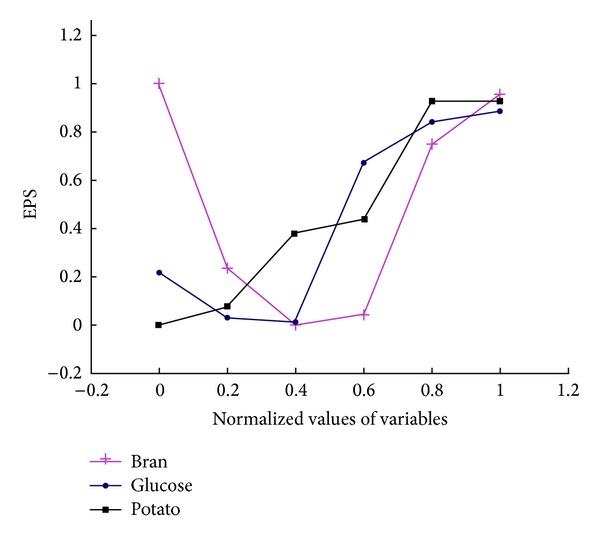
Predicted trend of EPS by ANN.

The artificial neural network with back-propagation algorithm was used to model the effect of media components such as potato, glucose, and bran on mycelial dry weight and EPS yield. The predictive trend of mycelial biomass and EPS was showed in Figures 3 and 4, respectively. The yield of mycelial dry weight with the increase of potato showed an increased-after-decreased trend in Figure 3 (black line). Meanwhile, the yield increased sharply with the increase of glucose (blue line). On the contrary, the yield of mycelial biomass decreased when the bran increased (red line). And Figure 4 showed the different predictive trends of EPS. The yield of EPS showed a trend of rising with both the increase of potato (black line) and that of glucose (blue line) in Figure 4. However, the yield showed a decreased-after-increased trend when the bran increased in the range of experiment (red line).

### 3.4. Verification of Predictive Model

According to the regression equation, the predicted maximum value of mycelial dry weight and EPS extraction was 1.047 g/100 mL and 0.367 g/100 mL, respectively, when the potato is 29.88 g/100 mL, the glucose is 1.01 g/100 mL, and the bran is 1.02 g/100 mL. To ensure the suitability of the model equation for predicting the optimum response values, experimental rechecking was performed using the recommended optimal conditions. It was found that the experimental value (0.914 ± 0.207 g/100 mL, 0.280 ± 0.123 g/100 mL, *n* = 3, resp.) was in agreement with the predicted one, indicating that the response surface model was suitable for optimizing the mycelial biomass and polysaccharide production.

### 3.5. Effect of ABM on Alcohol-Induced Liver Injury

**Figure 5:**
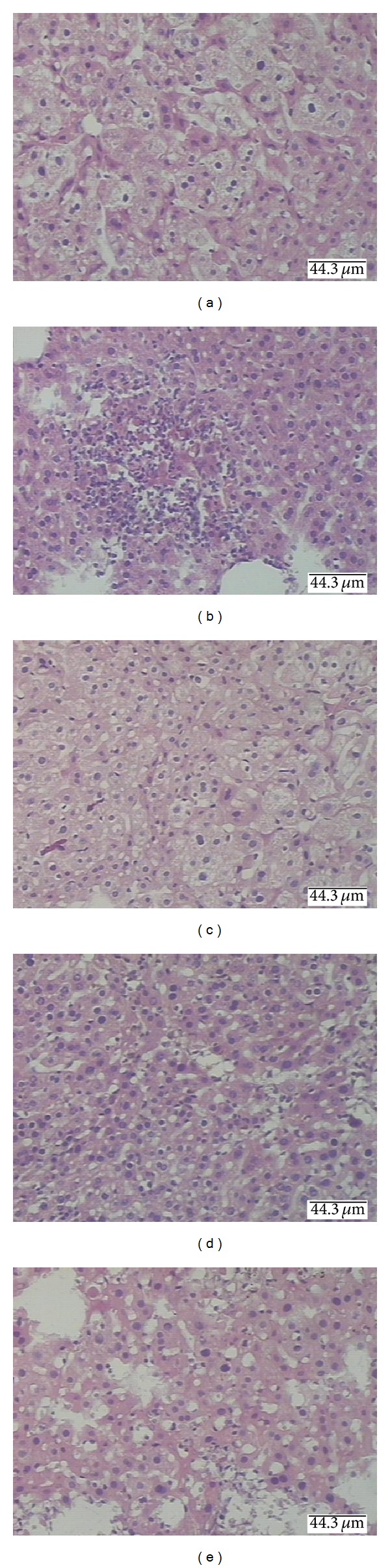
Histopathological analysis of mouse liver sections using hematoxylin and eosin staining. (a) Section from a normal control mouse liver. (b) The liver section that was obtained from alcohol-induced mice showed a variety of cavitation and necrosis in hepatocytes. (c) Liver tissue section prepared from the ABM-fp-treated group showed less cavitation and necrosis compared to (b). (d) Liver tissue section prepared from the ABM-fb-treated group showed less cavitation and necrosis. (e) Liver tissue section prepared from the ABM-fm-treated group showed less cavitation and necrosis compared to (b), but more than (c) and (d).

Ethanol-induced hepatic injury was indicated by liver pathological changes characterised by lymphocytes and neutrophils infiltration around the veins of hepatic tissues. In the blank control group (Figure 5(a)), there was no cavitation, necrosis, or fibrosis. The hepatocytes and plate from hepatic tissue sample had an intact structure, and the boundary between hepatocytes was clear. In contrast, the hepatocytes showed the hepatocytes' morphological damage in veins and the collection of lymphocyte and neutrophils in the ethanol control group (Figure 5(b)). This section displayed apparent cavitations in broad areas. However, the broad cavitations and the collection of lymphocyte and neutrophils in liver were somewhat attenuated in mice treated with ABM-fp (Figure 5(c)), ABM-fb (Figure 5(d)), and ABM-fm (Figure 5(e)). Compared with the ethanol control group, Figures 5(c) and 5(d) showed markedly fewer cavitations and less fibrosis in the liver. Yet, Figure 5(e) displayed few cavitations and moderate inflammatory changes. These experimental phenomena indicated that ABM could weaken or treat the liver injury caused by alcohol.

## 4. Conclusion

ABM as an edible and medicine mushroom is widely used in the world. And a variety of biological activities have been reported by several groups [29–33]. Several studies have actually found that the ABM extract could recover/repair the liver injury induced by CCl_4_ [34–36]. From this study, the maximal yield of mycelial biomass and extracellular polysaccharide was predicted at 1.047 g/100 mL and 0.367 g/100 mL, respectively, when the potato is 29.88 g/100 mL, the glucose is 1.01 g/100 mL, and the bran is 1.02 g/100 mL, using the response surface methodology. Simultaneously, the variation tendency was predicted by artificial neural network. Therefore, it is evident that the use of statistical method not only helped in locating the optimum levels of the most significant factors but also proved to be useful and satisfactory in this process-optimization. What is more, the recovery/reparative effects of ABM by submerged culture were observed through the hepatic histological sections of mice induced by alcohol. It indicated that ABM has a significant protective effect on alcohol-induced liver injury. And despite all this, some other experimental conditions are not included in the investigation and the application of ANN is not skilled. In addition, some related indicators of liver injury need to be further determined. Hence, further studies are still needed.

## Figures and Tables

**Table 1:** Variables and experimental design levels for response surface.

Variables (g/100 mL)	Coded symbols	Coded levels		
		−1	0	1
Potato	*X*_1_	15	20	25
Glucose	*X*_2_	1	2	3
Wheat bran	*X*_3_	1	2	3

**Table 2:** Design and experimental results of the four-factor Box-Behnken design.

Standard	Run order	*X*_1_	*X*_2_	*X*_3_	The yield of mycelial biomass (g/100 mL)		The yield of extracellular polysaccharide (g/100 mL)	
					Experimental	Predicted	Experimental	Predicted
4	1	25.00	3.00	2.00	1.033	1.038	0.268	0.264
9	2	20.00	1.00	1.00	0.761	0.761	0.367	0.367
2	3	25.00	1.00	2.00	0.882	0.877	0.285	0.289
3	4	15.00	3.00	2.00	0.852	0.857	0.230	0.226
8	5	25.00	2.00	3.00	0.936	0.931	0.243	0.247
5	6	15.00	2.00	1.00	0.764	0.769	0.320	0.316
6	7	25.00	2.00	1.00	0.945	0.950	0.358	0.354
7	8	15.00	2.00	3.00	0.755	0.750	0.205	0.209
11	9	20.00	1.00	3.00	0.710	0.720	0.252	0.244
16	10	20.00	2.00	2.00	0.841	0.841	0.264	0.264
14	11	20.00	2.00	2.00	0.841	0.841	0.264	0.264
13	12	20.00	2.00	2.00	0.841	0.841	0.264	0.264
1	13	15.00	1.00	2.00	0.701	0.696	0.246	0.246
9	14	20.00	2.00	2.00	0.841	0.841	0.264	0.264
10	15	20.00	3.00	1.00	0.912	0.901	0.318	0.326
15	16	20.00	2.00	2.00	0.841	0.841	0.264	0.264
12	17	20.00	3.00	3.00	0.902	0.902	0.235	0.235

**Table 3:** Analysis of variance (ANOVA) for the fitted quadratic polynomial model for optimization of biomass production and optimization of extracellular glucan production.

Source		Model	Lack of fit	Pure error	Corrected total
Sum of squares	Biomass	0.12	4.412*E* − 004	0.000	0.12
	EPS	0.031	2.643*E* − 004	0.000	0.031

D.f.	Biomass	9	3	4	16
	EPS	9	3	4	16

Mean square	Biomass	0.014	1.471*E* − 004	0.000	
	EPS	3.399*E* − 003	8.808*E* − 005	0.000	

*F*-value	Biomass	216.98	6.37		
	EPS	90.04	5.84		

Probability (*P* > *F*)	Biomass	<0.0001	0.0753 not significant		
	EPS	<0.0001	0.0528 not significant		

*R* ^2^: 0.9964, 0.9914; adj-*R* ^2^: 0.9918, 0.9804.
